
JOURNAL INFORMATION
==============================
NLM Title Abbreviation: ScientificWorldJournal
Journal ID: ScientificWorldJournal
NLM Journal ID: 101131163
Journal ID: TSWJ

The Scientific World Journal

ISSN: 2356-6140
EISSN: 1537-744X
Publisher: Wiley

ARTICLE INFORMATION
==============================
PMCID: PMC5848767
PMID: 18956115
DOI: 10.1100/tsw.2008.137
Article ID: 676579
Article version: 1
Subjects: Editorial

Acute Renal Failure from Adulteration of Milk with Melamine

Yang Vincent L.
Batlle Daniel *
Division of Nephrology and Hypertension, Department of Medicine, Northwestern University Feinberg School of Medicine, Chicago, IL, USA
*Daniel Batlle: d-batlle@northwestern.eduAcademic Editor: Graham V. Lees

Publication date: 2008
Electronic publication date: 2008 Oct 9
Volume: 8
First page: 974
Last page: 975
Received 2008 Oct 8; Revised 2008 Oct 9; Accepted 2008 Oct 9
Copyright: Copyright © 2008 Vincent L. Yang and Daniel Batlle.
Copyright year: 2008
License: This is an open access article distributed under the Creative Commons Attribution License, which permits unrestricted use, distribution, and reproduction in any medium, provided the original work is properly cited.
License URL: https://creativecommons.org/licenses/by/3.0/

==============================
Editorial
*TheScientificWorldJOURNAL* (2008) 8, 974–975
ISSN 1537-744X; DOI 10.1100/tsw.2008.137

TheScientificWorldJOURNAL
www.thescientificworld.com

### Acute Renal Failure from Adulteration of Milk with Melamine

Vincent L. Yang and Daniel Batlle*

*Division of Nephrology and Hypertension, Department of Medicine, Northwestern University Feinberg School of Medicine, Chicago, IL*

**E-mail**: d-batlle@northwestern.edu

*Received October 8, 2008; Revised October 9, 2008; Accepted October 9, 2008; Published October 9, 2008*

---

Every once in a while, unexpected pathologies and new diseases that afflict mankind are recognized and can be ascribed to a defined causative agent, be it infectious or chemical in nature. Recently, four deaths from presumed exposure to melamine, a material typically used in plastic manufacturing, in infant formula have been reported in China. Moreover, 50,000 illnesses resulting in about 12,000 hospitalizations have occurred[1]. The medical consequences resulting from contaminated milk and powdered formula have been noticed so rapidly that the impact cannot be appreciated fully. There is hardly any information published in scientific journals to assess the nature of this problem, but the lay press has been covering this issue widely during the last month.

The commentary by Drs. Lattupalli, Yee, and Kolluru is one of the few we can find in the scientific literature on this topic[2]. We thought this topic to be important and worth an editorial comment. To date, there are very few data on the nephrotoxic effects of melamine in humans. Much of the available information comes from the contamination of pet foods in 2007 that caused renal failure in cats and dogs. The culprit pet food was also manufactured in China and was found to contain melamine and cyanuric acid, which were believed to be the causative agents[3]. The pet food involved in the 2007 outbreak was also used to make animal feed, which prompted further testing and an official statement from the FDA stating that there was a very low risk to human health from consuming food from animals that ingested the tainted feed[4]. In fact, the toxic dose of melamine in rats is 3.1 g/kg (LD50) and cyuranic acid is 7.7 g/kg (LD50)[5]. This essentially classified either substance alone as nontoxic. However, when combined, the two substances form insoluble crystals that precipitate and can cause acute renal failure[6]. Subsequent studies on animals have shown that melamine, in particular in combination with cyanuric acid, causes deposition and precipitation of birefringent crystals[7]. Cyuranic acid, often used in pool chlorination, is allowed to be present in drinking water and animal feed, and is an intermediate byproduct of the bacterial metabolism of melamine[8]. This may explain the unexpected adverse events associated with the addition of melamine without the intentional addition of cyuranic acid. This has also prompted the FDA to revisit their stance on melamine and has led to the development of standard protocols to test for melamine and cyanuric acid in infant formula[9]. An updated statement by the FDA, released October 3rd, 2008, states that no amount of melamine in infant formula and a concentration of at most 2.5 ppm in other foods is acceptable[10].

The motive for the contamination of milk and powdered formula has been thought to be the high nitrogen content of melamine that, when added to formula, allows for dilution with water while maintaining the same apparent protein concentration. This egregious for-profit act exemplifies mankind's vulnerability to contaminated foods and the critical role the FDA and other food regulatory bodies around the world have in protecting the food supply. As we await more information from the unfortunate cases in

*Corresponding author.
©2008 with author.
Published by TheScientificWorld; www.thescientificworld.com

China to elicit the mechanism of renal failure in humans and the extent of this problem, we must continue our safeguards on all imports from countries with less regulation and more lax standards.

### REFERENCES

1. FDA update on Chinese infant formula contamination. http://www.modernmedicine.com/modernmedicine/Pediatric+News/FDA-updateon-Chinese-infant-formula-contamination/ArticleStandard/Article/detail/552475, September 24, 2008.
2. Lattupalli, R., Yee, J., and Kolluru, A. (2008) Nephrotoxicity of mala fide melamine: modern era milk scandal. *TheScientificWorldJOURNAL* **8,** 949–950.
3. Brown, C. et al. (2007) Outbreaks of renal failure associated with melamine and cyanuric acid in dogs and cats in 2004 and 2007. *J. Vet. Diagn. Invest.* **19(5),** 525–531.
4. Low Risk of Illness from Food Containing Melamine. http://www.fda.gov/consumer/updates/melamine051407.html. May 14, 2007.
5. Interim Melamine and Analogues Safety/Risk Assessment. http://www.cfsan.fda.gov/~dms/melamra.html. May 25, 2007.
6. Reimschuessel, R. et al. (2008) Evaluation of the renal effects of experimental feeding of melamine and cyanuric acid to fish and pigs. *Am. J. Vet. Res.* **69 (9),** 1217–1228.
7. Thompson, M.E. et al. (2008) Characterization of melamine-containing and calcium oxalate crystals in three dogs with suspected pet food-induced nephrotoxicosis. *Vet. Pathol.* **45**, 417–426.
8. Shelton, D.R. et al. (1997) Metabolism of melamine by klebsiella terragena. *Appl. Environ. Microbiol.* **63(7),** 2832–2835.
9. Turnipseed, S. et al. (2008) Determination of melamine and cyanuric acid residues in infant formula using LC-MS/MS. Laboratory Information Bulletin. LIB No. 4421. Volume 24. October.
10. FDA Issues Interim Safety and Risk Assessment of Melamine and Melamine-related Compounds in Food. http://www.fda.gov/bbs/topics/NEWS/2008/NEW01895.html. Oct 3, 2008.

**This article should be cited as follows:**

Yang, V.L. and Batlle, D. (2008) Acute renal failure from adulteration of milk with melamine. *TheScientificWorldJOURNAL* **8**, 974–975. DOI 10.1100/tsw.2008.137.
